# Gene expression profiling during the embryo‐to‐larva transition in the giant red sea urchin *Mesocentrotus franciscanus*

**DOI:** 10.1002/ece3.2850

**Published:** 2017-03-21

**Authors:** Juan Diego Gaitán‐Espitia, Gretchen E. Hofmann

**Affiliations:** ^1^ CSIRO Oceans & Atmosphere DivisionHobartTASAustralia; ^2^ Department of Ecology, Evolution and Marine BiologyUniversity of CaliforniaSanta BarbaraCAUSA

**Keywords:** developmental transcriptome, early ontogeny, echinoderm, gene expression, larvae, *Mesocentrotus franciscanus*

## Abstract

In echinoderms, major morphological transitions during early development are attributed to different genetic interactions and changes in global expression patterns that shape the regulatory program for the specification of embryonic territories. In order more thoroughly to understand these biological and molecular processes, we examined the transcriptome structure and expression profiles during the embryo‐to‐larva transition of a keystone species, the giant red sea urchin *Mesocentrotus franciscanus*. Using a de novo assembly approach, we obtained 176,885 transcripts from which 60,439 (34%) had significant alignments to known proteins. From these transcripts, ~80% were functionally annotated allowing the identification of ~2,600 functional, structural, and regulatory genes involved in developmental process. Analysis of expression profiles between gastrula and pluteus stages of *M. franciscanus* revealed 791 differentially expressed genes with 251 GO overrepresented terms. For gastrula, up‐regulated GO terms were mainly linked to cell differentiation and signal transduction involved in cell cycle checkpoints. In the pluteus stage, major GO terms were associated with phosphoprotein phosphatase activity, muscle contraction, and olfactory behavior, among others. Our evolutionary comparative analysis revealed that several of these genes and functional pathways are highly conserved among echinoids, holothuroids, and ophiuroids.

## 1 Introduction

Indirect development is considered an apomorphic, or derived, life‐history mode in marine metazoans (Raff, 2015). In this developmental mode, embryogenesis promptly forms an intermediate larval stage that nourishes the proliferation of undifferentiated multipotent cell precursors in charge of postembryonic adult formation (Arenas‐Mena, Wong, & Arandi‐Foroshani, 2007). During the transition from embryo to larval stage, the interaction of multiple genetic regulatory networks (GRNs) determines the patterns of gene activity and differentiation of developmental modules in marine invertebrates (Raff & Sly, 2000). In echinoderms, for example, the unique combination of regulatory genes (e.g., transcription factors) in embryonic space and time contribute to shaping the regulatory program for larval skeletogenesis (Dylus et al., 2016; Gao & Davidson, 2008), endomesodermal (Peter & Davidson, 2010), and ectodermal specification (Nakata & Minokawa, 2009). Despite the differences in larval development in this group (e.g., pluteus‐, bipinnaria‐, and auricularia‐like larvae), comparisons of their GRN architectures have detected highly conserved orthologous regulatory genes among the extant echinoderm classes (Hinman & Davidson, 2003a,b; Hinman, Nguyen, Cameron, & Davidson, 2003; Hinman, Nguyen, & Davidson, 2003). While the network logic employed is the same in these organisms, the transcription factors underlying certain functions have been the subject of evolutionary change, resulting in gene duplications, protein function diversification, and regulatory genes co‐opted to different functions (Dylus et al., 2016; Hinman & Davidson, 2007; Hinman, Nguyen, Cameron, et al., 2003; McCauley, Weideman, & Hinman, 2010; McCauley, Wright, Exner, Kitazawa, & Hinman, 2012).

From an ecological and evolutionary perspective, the process of development could be considered as a balancing act in an unpredictable world. It is often remarkably resilient, producing consistent phenotypes in the face of new mutations and environmental perturbations, but it is also adaptable, allowing for the evolution of novel phenotypic traits in response to environmental change (Garfield et al., 2013; Sultan, 2007) For example in sea urchins with planktotrophic life‐history mode, larvae are developmentally plastic in response to changes in environmental conditions (Byrne, Lamare, Winter, Dworjanyn, & Uthicke, 2013; Padilla‐Gamiño, Kelly, Evans, & Hofmann, 2013; Yu et al., 2013) and food availability (Adams, Sewell, Angerer, & Angerer, 2011; Carrier, King, & Coffman, 2015; McAlister, 2008), which allow them to regulate the rate of growth and skeletal formation. Such developmental adjustments are achieved mostly through variation in the expression of regulatory genes (Carrier et al., 2015; Runcie et al., 2012). This kind of variation has been documented as an intrinsic characteristic of natural populations of sea urchins (e.g., *Strongylocentrotus purpuratus*), in which changes in timing or level of gene expression are well buffered during early development by on/off switch‐like regulatory mechanisms, while during later development they have a greater impact on the expression of downstream target genes and on morphology (Garfield et al., 2013).

Most of the efforts to understand GRN architectures and changes in the expression of regulatory and structural genes during indirect development have used the purple sea urchin *S. purpuratus* as a study system (Garfield et al., 2013; Hammond & Hofmann, 2012; e.g., Hinman & Davidson, 2003a; Rafiq, Cheers, & Ettensohn, 2012; Rafiq, Shashikant, McManus, & Ettensohn, 2014; Runcie et al., 2012; Tu, Cameron, & Worley, 2012). However*,* thanks to the advances in next‐generation sequencing (NGS) technologies, the ability to sequence the entire transcriptome of a given tissue or life‐history stage in a matter of days is providing new opportunities to explore the complexity of developmental GRNs in other echinoderms (Delroisse, Ortega‐Martinez, Dupont, Mallefet, & Flammang, 2015; Dilly, Gaitán‐Espitia, & Hofmann, 2015; Dylus et al., 2016; Gildor, Malik, Sher, Avraham, & Ben‐Tabou de‐Leon, 2015; Tulin, Aguiar, Istrail, & Smith, 2013) and marine invertebrates (Jackson & Degnan, 2016; Layden, Rentzsch, & Röttinger, 2016). As changes in gene expression may underlie many of the phenotypic differences between species (Brawand et al., 2011), studying transcriptomic divergence of sympatric species may shed light upon the initial genetic targets of natural selection in speciation events (Filteau, Pavey, St‐Cyr, & Bernatchez, 2013). Under this context, here we examine the transcriptome structure and the expression of genes during the embryo‐to‐larva transition of the giant red sea urchin *Mesocentrotus franciscanus* (syn. *Strongylocentrotus franciscanus*), exploring questions regarding the level of conservation of homologous genes and functional pathways. This sea urchin is one of the most distantly related strongylocentrotid species yet lives in sympatry with *S. purpuratus* (Lee, 2003), and the development, survival, and settlement of its planktotrophic larvae are affected by changes in environmental conditions and by large‐scale oceanographic processes associated with “El Niño” (Dewees, 2003; Ebert, Schroeter, Dixon, & Kalvass, 1994). Therefore, assessing the developmental genetic background of early ontogenetic stages of *M. franciscanus* would provide valuable genomic and molecular tools to study not just the evolution of GRNs in echinoderms, but also the plastic and adaptive capacity of these organisms to respond to the rapid environmental change projected for the future in one of the most productive marine regions in the world, the California Current Large Marine Ecosystem (Hofmann et al., 2014).

## 2 Materials and Methods

### 2.1 Ethics statement

This study was carried out in strict accordance with the recommendations in the Guide for the Care and Use of Laboratory Animals of the Comisión Nacional de Investigación Científica y Tecnológica de Chile (CONICYT). The protocol was approved by the Committee on the Ethics of Animal Experiments of the University of California, Santa Barbara.

### 2.2 Animal collection, fertilization, and larval culturing

Mature adults of the giant red sea urchin (*Mesocentrotus franciscanus*) were collected by SCUBA divers near Goleta Pier in the Santa Barbara Channel, California, USA (34°24′N, 119°49′W) in June 2013. Animals were maintained in a flowing seawater system under ambient conditions (~14°C, 32%o and pH ~ 8.0) at the University of California, Santa Barbara. Gametes were obtained by intracoelomic injection of 0.5 mol/L KCl. The eggs were resuspended in 0.35‐μm‐filtered, UV‐sterilized seawater (FSW) at ambient temperature and pCO_2_. Sperm was collected dry and kept on ice until dilution on FSW prior to fertilization. Nine families were established using single dam‐sire crosses (the sperm from one male was used to fertilize the eggs of a single female). After fertilization, embryos from each family were equally (~20 embryos/ml) and randomly distributed in plastic buckets. Development was tracked by recording the proportion of embryos to reach gastrula and early pluteus stages (~29 hr and 82 hr, respectively). At these ontogenetic stages, larvae from each bucket were collected in a small water volume using a reverse‐filtration siphon and transferred to 1.5‐ml Eppendorf tubes. These samples were then centrifuged at low speed to pellet the larvae, removing the excess water and adding 1 ml of TRIzol Reagent (Invitrogen, Carlsbad, CA, USA). Samples were quickly frozen in liquid nitrogen and stored at −80°C for subsequent analysis.

### 2.3 RNA extraction, cDNA library construction, and sequencing

Total RNA was extracted from each larval culture (~30,000 larvae) at gastrula and early pluteus stages (18 samples) following the guanidine isothiocyanate method (Chomczynski & Sacchi, 1987). Extracted RNA was further cleaned to remove degraded, fragmented RNA using an RNeasy® Mini Kit (Qiagen, Valencia, CA) according to manufacturer instructions. Quality and quantity of the RNA were analyzed by Agilent 2100 Bioanalyzer RNA assays and evaluated by calculating the ratio of the 28S and 18S ribosomal RNA intensity peaks. High‐quality RNA (RINs over 8.5) was pooled (~5 μg each sample) according to their ontogenetic stages (gastrula and early pluteus) in order to average out differences in expression levels between families. Illumina TruSeq RNA library preparation, clustering, and sequencing reagents were used throughout the process following the manufacturer's recommendations (Illumina, San Diego, CA). Briefly, 1 μg of total RNA was used as starting material for library preparation with the Illumina TruSeq RNA preparation Kit V2. Poly‐T oligo‐attached magnetic beads were used to purify mRNA, which was then fragmented for 8 min at 94°C. First‐strand and second‐strand DNA were subsequently synthesized. Libraries were multiplexed with Illumina barcodes and sequenced in two lanes at GENEWIZ, Inc. (South Plainfield, NJ) using a 2 × 100 paired‐end (PE) configuration on Illumina HiSeq2000 platform. Image analysis and base calling were conducted using the HiSeq Control Software (HCS). These base calls were used to generate BCL and FASTQ files by mean of the Illumina's CASAVA 1.8.2 program.

### 2.4 Transcriptome assembly and annotation

Following sequencing, quality control of the raw data was performed using the CLC Genomics Workbench software v.8.5 (CLC bio, Denmark) in a four‐step pipeline: (1) removal of adapter sequences; (2) removal of all reads containing more than 5% of ambiguous nucleotides (“N”); (3) trimming base pairs with a Phred quality score ≤ 30 from the 3′‐end of each sequence; and (4) removal of reads shorter than 30 bp after trimming. Because the available reference genome of *M. franciscanus* is still in the early stages (low coverage and annotations), three de novo assemblies were developed in this study: (1) the reference transcriptome including all the libraries and ontogenetic stages; (2) the gastrula; and (3) the pluteus transcriptomes. Assemblies of high‐quality reads were carried out using the Trinity software (Grabherr et al., 2011). Assemblies were performed with default settings and a minimum contig length of 200 nt. Reads that were not incorporated into any contig (i.e., singletons) were discarded and excluded from further analyses. Duplicate sequences were removed after the de novo assemblies.

The de novo assemblies were separately blasted against the UniProt (Swiss‐Prot and TrEMBL) and NCBI RefSeq (nr) protein databases using the BLASTX algorithm with an e‐value cutoff of 10 e^−5^. Annotated unigenes (consensus, nonredundant sequences) were further searched for Gene Ontology (GO) terms using the Blast2GO software (Conesa et al., 2005) according to the main categories of Gene Ontology (GO; molecular functions, biological processes, and cellular components; Ashburner et al., 2000). Complementary annotations were performed with the InterProScan v.5 software (Jones et al., 2014), which provides functional analysis of proteins by classifying them into families and predicting domains and important sites. The annotation results were further fine‐tuned with the Annex and GO slim functions of the Blast2GO software in order to improve and summarize the functional information of the transcriptome dataset. The distribution of annotated unigenes among GO categories was mapped using the WEGO software (Ye et al., 2006). Additionally, a GO enrichment analysis using Fisher's exact test was also performed in Blast2GO to test whether any of the GO terms appeared significantly over‐ or underrepresented in a pairwise comparison between the two ontogenetic stages. Finally, a comparison of overall nucleotide sequence homology between the gastrula and pluteus transcriptome of *M. franciscanus* and the genome of *S. purpuratus* was completed using the Kyoto Encyclopedia of Genes and Genomes (KEGG) and its automated assignment server (KAAS; Moriya, Itoh, Okuda, Yoshizawa, & Kanehisa, 2007).

### 2.5 Comparison of gastrula and pluteus transcriptomes among echinoderms

In order to explore the level of evolutionary conservation of gene/protein functions in the gastrula and pluteus stages of sea urchins and their relatives, we compared our de novo assembled transcriptomes of *M. franciscanus* with the those of the sea urchins *S. purpuratus* and *Lytechinus variegatus*, the sea cucumber *Parastichopus parvimensis* (gastrula), and the brittle star *Amphiura filiformis* (pluteus). The raw unassembled datasets of these species were downloaded from the NCBI SRA database under the accessions SRX120411‐SRX120412 (*S. *purpuratus), SRX766170‐SRX766173 (*L. variegatus*), SRX146991 (*P. parvimensis*), and SRX666715 (*A. filiformis*). The same quality control and pipelines applied for *M. franciscanus* were implemented to obtain high‐quality contigs of the additional species. Transcripts of the five species were translated and used for comparisons of orthologous clusters with the OrthoVenn software (Wang, Coleman‐Derr, Chen, & Gu, 2015) using default parameters to identify GO categories and any GO enrichment.

### 2.6 Differential gene expression analysis

In order to identify differentially expressed genes on early developmental stages of the giant red sea urchin *M. franciscanus*, reads of each sample were mapped to the de novo assembled developmental transcriptome (gastrula + pluteus) using CLC Genomics Workbench software v.8.5 (CLC bio, Denmark). The length fraction was set to 0.7, and the minimum similarity was set to 0.9, which meant that at least 70% of the individual reads had at least 90% identity with the reference sequences to be mapped and aligned. The read counts were normalized by calculating the number of reads per kilobase per million mapped reads (RPKM) and log_2_ transformed. Then, the Manhattan metric distance was used for hierarchical cluster analysis, and a Kal's test was used to compare gene expression levels for larval stages. The Kal's test relies on an approximation of the binomial distribution by the normal distribution. This proportion‐based test for gene expression is applicable for single sample to single‐sample comparisons. The cutoff of FDR *p*‐value correction ≤0.05 and fold‐change value ≥2 was used to determine significant differential expression. The results of this comparative analysis were checked by inspecting the distribution of differentially expressed genes using volcano plots. Enrichment analysis of differentially expressed genes was conducted by hypergeometric tests using CLC Genomics Workbench software v.8.5 (CLC bio, Denmark) against the background of expressed genes (cutoff P_FDR_ ≤ 0.05). The REViGO Web server was used for visualization of the GO terms associated with the differentially expressed genes. Treemaps in REViGO present hierarchical data as nested rectangles and provide an intuitive visualization of the dataset (Supek, Bošnjak, Škunca, & Šmuc, 2011). Size of the rectangles was adjusted to reflect the *p*‐value using the abs_log_pvalue option in REViGO. Finally, comparison of the expression profiles during the embryo‐to‐larva transition of *M. franciscanus* and its sympatric species *S. purpuratus* was developed using the Query tool available in Echinobase (echinobase.org; Cameron, Samanta, Yuan, He, & Davidson, 2009; Tu, Cameron, & Davidson, 2014). Here, our DEGs were assigned to the 24 Function Classes described in Tu et al. (2014) and compared to the profile of those embryonic genes in *S. purpuratus*.

## 3 Results and Discussion

### 3.1 Sequence analysis and assembly

A total of 53.85 gigabases (Gb) of sequence were generated from four libraries of *M. franciscanus* during early developmental stages. The number of raw reads among libraries ranged between 81.3 and 119.1 million (mean = 102.2 million, *SD* = 18.1 million), showing similar quality scores (mean Q‐scores = 35.6; mean Q‐scores ≥ 30 = 91.5%; Table S1). The SRA raw reads were deposited on GenBank public database under the accession numbers SRS823202 (gastrula), SRS823216 (gastrula) SRS823218 (pluteus), and SRS823221 (pluteus) of the bioproject PRJNA272924. After a stringent filtering process, ~91% high‐quality, adapter‐free, and nonredundant reads were retained for de novo assembly and further downstream analyses. Quality metrics of assembled transcriptomes (e.g., N_50_ and L_50_) are summarized in Table 1. Completeness assessments using CEGMA identified 247 out of the 248 core proteins (99.6%) as complete (defined as >70% alignment length with core protein) and 248 (100%) as partially present (Table 1). This was consistent with the Benchmarking Universal Single‐Copy Orthologs (BUSCO; Table 1).

**Table 1:** Statistics of de novo assembled transcriptomes of *Mesocentrotus franciscanus*

Assembly details	Reference	Gastrula	Pluteus
	Trinity	Trinity	Trinity
Number of contigs	176,885	136,968	119,470
Number of contigs > 1 kb	34,221	26,944	30,225
Total assembled nucleotides	140,304,105	106,291,147	113,007,730
Average GC content (%)	39.88	39.74	40.34
Longest contig length (bp)	36,388	29,131	38,340
Average contig length (bp)	793.2	776.03	945.9
N_50_ (bp)	1,637	1,479	1,992
L_50_	21,423	17,978	15,304
CEGMA complete (%)	99.6	98.4	98.8
CEGMA partial (%)	100	100	99.6
Average number of orthologs per CEG	2.15	2.12	2.02
BUSCO completeness (%)	88	85	87
BUSCO fragmented (%)	4.6	6.5	4.4
BUSCO missing (%)	7.2	7.6	8.1

### 3.2 Functional annotation

Our reference de novo assembled transcriptome contains 176,885 tentative consensus sequences with an N_50_ of 1,637 bp and an L_50_ of 21,423 (Table 1). From these unigenes, 60,439 (~34%) were blasted to known proteins in the public databases NCBI (nr) and UniProt (Swiss‐Prot and TrEMBL), while 116,446 (~66%) had no matches and may represent: (1) specific unigenes of *M. franciscanus* with unknown function; (2) sequences with low similarity to those compared in public databases; and/or (3) chimeric sequences. Although the percentage of unigenes with a BLAST‐hit may appear to be relatively low, we found that the number of unigenes with significant alignments (≤1 e^−5^) to known proteins in *M. franciscanus* is higher than those reported in other studies with nonmodel echinoderms (Delroisse et al., 2015; Dilly et al., 2015; Gaitán‐Espitia, et al., 2016; Gillard, Garama, & Brown, 2014; Pérez‐Portela, Turon, & Riesgo, 2016; Stewart, Stewart, & Rivera‐Posada, 2015; Vaughn, Garnhardt, Garey, Thomas, & Livingston, 2012; Zhou et al., 2014). Most of the annotated unigenes hit against the purple sea urchin *Strongylocentrotus purpuratus* (88.4%), followed by the acorn worm *Saccoglossus kowalevskii* (1.5%), and the Pacific oyster *Crassostrea gigas* (<1%; Fig. S1).

In order to obtain a comprehensive insight into the possible functions of blasted unigenes in the gastrula (48,347) and pluteus (44,977) transcriptomes, we merged the gene ontology (GO) annotations obtained from Blast2GO and InterProScan, resulting in 107,758 and 98,586 GO terms, respectively. These GO annotations were similarly distributed among the main GO categories in both transcriptomes (Table S2) with a higher representation of biological process (BP, 46.2%: 47.8%), followed by cellular components (CC, 27.3%: 28.5%) and molecular functions (MF, 26.5%: 23.7%). In BP, most of the GO terms were grouped into major subcomponents like cellular process (GO:0009987), metabolic process (GO:0008152), and pigmentation (GO:0043473; Figure 1). For CC, the highest percentage of GO terms was associated with cell (GO:0005623) and organelle (GO:0043226), while binding (GO:0005488) and catalytic activity (GO:0003824) were the main subgroups in the MF category (Fig. S2). Further comparisons of unigenes and GO terms between the gastrula and the pluteus transcriptomes revealed 13,927 shared clusters of proteins (containing both developmental stages) and 4,665 single‐copy gene clusters (Figure 2a). Shared clusters showed significant enriched GO categories associated with tissue development, segmentation, morphogenesis, among other biological processes (Table S3). This is explained by the higher representation of Toll‐like receptor proteins (e.g., sp‐Tlr037), binding proteins (e.g., Sp‐Pacsin2), developmental proteins such as the Frizzled proteins Frizz4 and the Dishevelled proteins Dsh involved in the Wnt signaling pathway (Bilić et al., 2007), and proteins from the TFG‐beta family involved in growth such as the Admp (Table S4). Unique clusters in the gastrula transcriptome (Table S5) showed 34 enriched GO categories (Table S4), most of them linked to sensory perception, cell fate specification, and postembryonic development. In the pluteus transcriptome, on the other hand, despite the presence of unique clusters associated with developmental processes (e.g., system development, GO:0007399; cell migration, GO:0016477; morphogenesis of an epithelium, GO:0002009; somatic muscle development, GO:0007525; Table S6) only three enriched GO categories were detected (Table S4).

**Figure 1:**
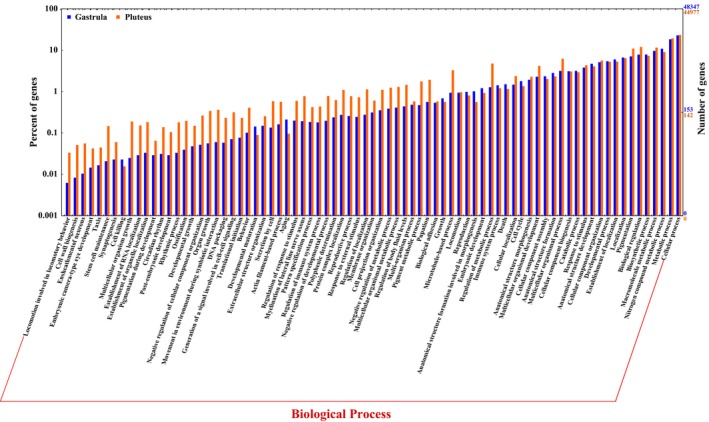
Gene Ontology categories for biological processes in the gastrula and pluteus transcriptomes of *Mesocentrotus franciscanus*. Categories are shown on the *x*‐axis; while the right *y*‐axis indicates the number of genes per category, the left *y*‐axis indicates the percentage of genes in the main category

**Figure 2:**
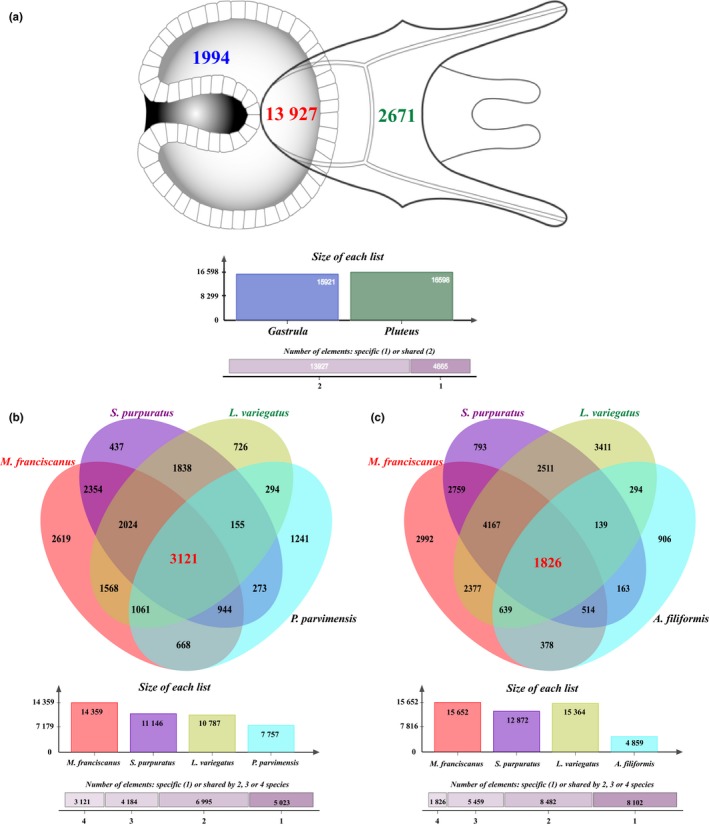
Protein clusters among early developmental transcriptomes of echinoderms. (a) Comparison between gastrula and pluteus stages of *Mesocentrotus franciscanus*. Analysis of orthologous clusters among gastrulas (b) and pluteus (c) stages of the sea urchins *M. franciscanus*,* Strongylocentrotus purpuratus* and *Lytechinus variegatus*, the sea cucumber *Parastichopus parvimensis* (gastrula), and the brittle star *Amphiura filiformis* (pluteus)

**Figure 3:**
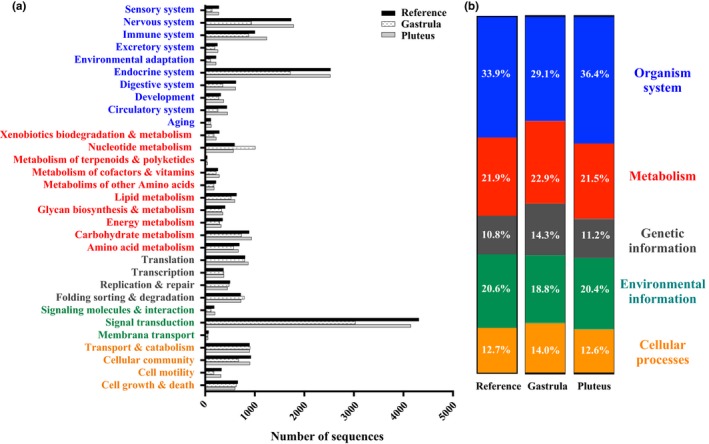
Unigenes homology to KEGG genes obtained from the KAAS server. (a) Number of sequences assigned to each subcategory of the reference hierarchy KOs. (b) Percentage distribution of the five top KEGG orthology categories in the reference, gastrula, and pluteus transcriptomes of *Mesocentrotus franciscanus*

Active biological pathways during early ontogenetic stages of *M. franciscanus* were obtained from the KEGG Orthology database. Here, a total of 5,428 transcripts were linked to EC numbers and mapped to six enzyme classes in which hydrolases showed the highest number of sequences followed by transferases and oxidoreductases (Fig. S3). This pattern was consistent among the three *do novo* assemblies (Fig. S3). The high representation of these three main enzyme classes could be related to particular developmental processes. For instance, some hydrolases (e.g., lysosome acid hydrolases) are known to play an important role in intracellular digestion and synchronic degradation of the major yolk glycoproteins (e.g., vitellins) during early development of sea urchins (Kari, 1985; Yokota & Kato, 1988). Furthermore, some transferases (e.g., glycosyltransferases) are characterized by changes in their activity after fertilization of sea urchin eggs, regulating development by allowing migration of particular groups of cells destined to give rise to the digestive, skeletal, and nervous systems (Evans & Bosmann, 1977). Likewise, oxidoreductases such as the malate dehydrogenase (EC 1.1.1.37) have been documented to increase their catabolic activity before the onset of gastrulation and reach high levels later during embryogenesis differentiation of echinoderms (Ozaki & Whiteley, 1970; Swezey & Epel, 1988). Posterior KEGG analysis, using KAAS, retrieved 6,218 KO identifiers for 22,081 unigenes (~37% of annotated sequences) in 225 KEGG pathways. From these, signal transduction, endocrine system, and nervous system were the most represented pathways in the reference, gastrula, and pluteus transcriptomes (Figure 3a), and unigenes were mainly allocated to the KO categories of Organism System, Metabolism and Environmental Information Processing (Figure 3b).

### 3.3 Identification of genes expressed during the embryo‐to‐larva transition

In developmental biology, gene regulatory networks (GNRs) are used to describe the progression of regulatory states, in embryonic space and time, necessary to specify different cell types present in a multicellular organism (Davidson, 2006). For sea urchins, these GRNs are represented by over 200 experimentally verified regulatory interactions acting during early development, from the unfertilized egg through the formation of a planktonic feeding larva (Israel et al., 2016). In our study, we identified ~2,600 functional, structural, and regulatory genes in *M. franciscanus* involved in developmental process (GO:0032502). Some of these genes (~580) were specifically related to several aspects of embryonic development (GO:0009790), from organogenesis through to the differentiation of the musculoskeletal, cardiovascular, and nervous system (Table S7). As has been documented in other studies, sea urchin fertilization triggers a series of preprogrammed events functioning to activate egg metabolism, incorporate the paternal genome, and initiate development (Walker, Unuma, & Lesser, 2006). Perhaps one of the first events is the rise of calcium signals (Ca^2+^), which are modulated by ryanodine receptors genes (RyRs) in order to prevent polyspermy and reinitiate the cell cycle for development (Jaffe, Giusti, Carroll, & Foltz, 2001). Then, other events related to membrane depolarization, cell proliferation, cell migration, and cleavage formation are activated by genes such as the nodal precursor (NODAL), the phosphatidylinositol‐triphosphate 3 phosphatase (PTEN), the Ras‐related c3 botulinum toxin (RAC1), the Nck‐associated protein 1 (NCKAP1), and the Slit homolog 3 protein (SLIT3; Table S7; Crabbe & Vleminckx, 2008; Ueno, Kono, & Iwao, 2006). Once the fertilized egg begins to divide, several genetic cascades, involving growth factors (e.g., MEGF8, EGF, AP2M1, FIBPB, TGFBI, GRB2, FGFR‐3, HGF, VEGF, PDGF‐D), take place to induce embryonic pattern specification (GO:0009880), regionalization (GO:0003002), and developmental maturation (GO:0021700; Table S7; Cross & Dexter, 1991). During these processes, additional signaling pathways, including Wnt signaling, play important roles in regulating axis formation and nervous system patterning by the establishment of ternary complexes with low‐density lipoprotein receptors (e.g., LRP6) and Frizzled receptors (e.g., FZD5; Table S7; Bilić et al., 2007). After Wnt stimulation, the segment polarity protein disheveled (e.g., DVL3) mediates the phosphorylation of the LRP6 by Casein kinases (e.g., CSK1D), promoting recruitment of the negative regulator Axin (e.g., AIDA‐A), which, in turn, stabilizes Wnt signaling transducers such as the β‐catenin (Table S6; Angerer & Angerer, 2000; Bilić et al., 2007).

Between the early blastula and early gastrula stages of sea urchins, all of the GRNs controlling the major embryonic territories (i.e., skeletogenic, endomesoderm, and ectoderm) have been activated, initiating the programs of cell‐type‐specific differentiation (Wei, Angerer, & Angerer, 2006). These processes are modulated by subcircuits of genes encoding transcription factors and their linkages (Israel et al., 2016). In our study, we were able to identify genes related to transcription regulatory activity (91:420; GO:0030528), transcription factor complex (143:533; GO:0005667), and transcription factor binding (249:241; GO:0008134) in the gastrula and pluteus transcriptomes (Table S8). The disparity in the number of genes for some of these GO terms between both transcriptomes (Pearson Chi‐square test, *p* < .05; Table S2) could reflect differences in the time of specification and differentiation of cell types (e.g., endomesoderm vs. neural cell types), and also sequential activation of genes in a regulatory hierarchy (Wei et al., 2006). In fact, in our analysis, some genes related to the determination of left/right symmetry (GO:0007368) were only present in the pluteus transcriptome (Table S7), supporting the idea of time‐dependent processes associated with GRNs (Israel et al., 2016). Overall, the most abundant transcription factors in early stages of *M. franciscanus* were associated with the FOX (Forkhead box), E2F, and HES (Hairy and enhancer of split) families, followed by the CCR4‐NOT complex, the TFIIA (transcription initiation factor iia), and the CREB (cAMP response element‐binding protein) proteins (Table S8 and Fig. S4). Other transcription factors in lower frequency but similarly represented in both transcriptomes were SOX, TFIIH, TCF21, RUNX2, GTF3A, MEF2A, HIF1A, and SUM‐1 (Table S8 and Fig. S4). However, some developmental regulators were more represented in particular stages. For instance, in the gastrula transcriptome, a higher number of genes linked to HLF (hepatic leukemia factor), GATA, DNMT1 (DNA [cytosine‐5]‐methyltransferase 1), MAPK (mitogen‐activated protein kinase), RIBIN (rrna promoter binding protein), and KDM1A (lysine‐specific histone demethylase 1), were found in comparison with the fully completed embryo (Table S8 and Fig. S4). In the pluteus transcriptome, on the other hand, genes related to ETS (E26 transformation‐specific), HNF (hepatocyte nuclear factor), RBFOX1 (RNA‐binding protein fox), ING4 (inhibitor of growth protein 4), ERG (transcriptional regulator erg), TEAD1 (transcriptional enhancer factor tef‐1), ZF‐TFs (zinc finger), homeobox, and T‐box proteins were more abundant than in the gastrula transcriptome (Table S8 and Fig. S4). In conjunction, the expression of these genes and other developmental regulators determine the progressive changes in form that characterize the embryo‐to‐larva transition in sea urchins (Dylus et al., 2016; Israel et al., 2016; McCauley et al., 2010; Rafiq et al., 2012, 2014; Wei et al., 2006).

### 3.4 Evolutionary conservation of genes among echinoderms

The embryogenesis and early larval development of echinoderms involve the interactions of regulatory, structural, and functional genes that, in some cases, are highly conserved even among distantly related echinoderm species (Hinman & Davidson, 2003a,b; Hinman, Nguyen, Cameron, et al., 2003; Hinman, Nguyen, & Davidson, 2003). However, differences in skeleton formation and embryonic territories specification in this group are the result of evolutionary changes, which include gene duplications, protein function diversification, and genes co‐opted to different functions (Dylus et al., 2016; Hinman & Davidson, 2007; Hinman, Nguyen, Cameron, et al., 2003; McCauley et al., 2010, 2012). Here, comparisons of early developmental transcriptomes of echinoderms revealed several proteins/genes that are highly conserved among sea urchins, sea cucumbers, and brittle stars (Figure 2b,c). Although the number of proteins from each species was very similar (Table S9), more orthologous clusters were detected within Echinoidea in comparison with the other echinoderms (Figure 2b,c; Table S9), which is expected because of their closer evolutionary history (Dilly et al., 2015). Overall, 3,121 orthologous protein clusters were shared between the gastrulas of echinoids and holothuroids (Figure 2b). These clusters were mainly represented by proteins related to the neuron navigator Nav3l, the general transcriptional corepressor trfA, the RNA‐binding Rbms3, the phospholipase C‐like 2 (Plcl2), the cyclin E (CycE), some transcription factors (e.g., KEN1, RFX‐DAF19, GTF2B, GTF2H2, UNC86, KAY, TBX1, bZIP8, ASCL, UNC30, AMOS), and some adhesion proteins (e.g., Agrin; Table S10). Comparison among gastrulas of *M. franciscanus*,* S. purpuratus,* and *L. variegatus* revealed 7,784 orthologous clusters that are unique for echinoids (Figure 2b). From these clusters, 2,024 were shared among the three species and included a group of transcription factors (e.g., CHE‐1, TFIIH, FOXO, ADF‐1, NFYB), proteins related to segmentation polarity homeobox, hedgehog signaling pathway, developmental processes (e.g., segment polarity protein disheveled Dsh, protein lap4, Cramp1L, Klf11, Pax2), and other cellular components and molecular functions (Table S11). Sympatric species such as *M. franciscanus* and *S. purpuratus* shared 2,354 orthologous clusters (Figure 2b). Most of these clusters were associated with ion binding (GO:0043167), metabolic process (GO:0008152), biological regulation (GO:0065007), and developmental process (GO:0032502; Table S12). Some clusters between *M. franciscanus* and *S. purpuratus* were particularly associated with sea urchin embryogenesis, involving homolog proteins of the blastula protease 10 (Adam/TsL6), the short gastrulation Chordin, and the protein twisted gastrulation (Tsg; Table S12).

Exploring the pluteus transcriptomes of sea urchins and brittle stars allowed us to identify 1,826 orthologous clusters between these two groups (Figure 2c). Most of the proteins in these clusters were linked to eukaryotic translation initiation factor 3 subunit D (Eif3d), neuron navigator Nav1/2/3, kinesin protein KifC3L2, calcium‐activated potassium channel Kncma1, axonemal dynein Axndhc3h, Cadherin4L, several zinc finger proteins (e.g., Z156, Z35), growth factor receptor (Grb2), and transcription factors (e.g., TFIIB/GTF2b, TfIID, SOX, KEN2, GTF3C5, AP‐1, ELT‐2, KAY; Table S13). The presence of several ophiuroid orthologs of sea urchin embryonic development (Vaughn et al., 2012) and the remarkable resemblance in the expression of regulatory genes between both groups (McIntyre, Lyons, Martik, & McClay, 2014) suggest a similar molecular makeup of their major embryonic territories (i.e., skeletogenic, endomesoderm, and ectoderm; Dylus et al., 2016). Within echinoids, 11,814 orthologous clusters were identified as unique for sea urchins, from which 4,167 were shared among *M. franciscanus*,* S. purpuratus,* and *L. variegatus* (Figure 2c). Proteins in these clusters were mostly assigned to the immune system (e.g., Toll‐like receptor Tlr083), hydrolase activity (e.g., AcheL_4), the inhibitor of growth family (e.g., Ing4), DNA binding (e.g., RtL58), cellular protein modification process (e.g., tubulin tyrosine ligase Ttl5), nucleotide binding (e.g., Cugbp1, PolppL_12), ectoderm and mesoderm development (e.g., Snail), cell differentiation (e.g., Cchcr1), and transcription factors (e.g., ERG, HAMLET, HESD, GRAUZONE, NFYB, GAGA, RUN, UNC‐3, and FOXJ2; Table S14). In addition, 2,759 clusters were shared between pluteus of the sympatric species *M. franciscanus* and *S. purpuratus* (Figure 2c). Clusters between these Californian sea urchins showed similar distribution of proteins compared to their gastrulas (Table S12), with the addition of clusters related to mesodermal cell fate commitment (e.g., Forkhead box B), anterior/posterior axis specification (e.g., Nanos, Pkn kinase), embryonic ectoderm and endoderm development (e.g., Eed, Nlk), embryonic body morphogenesis (e.g., Z298, Ptprr), and embryonic digestive tract development (e.g., Protein LAP1/Traf3; Table S15).

### 3.5 Gene expression profiles and clustering

**Figure 4:**
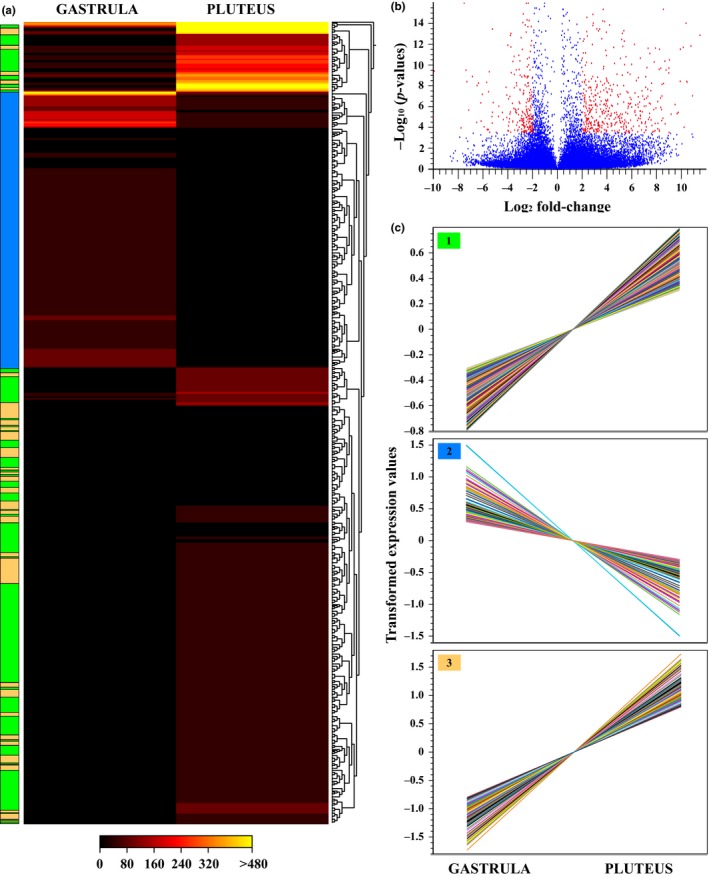
Gene expression profiles during early development of *Mesocentrotus franciscanus*. (a) Heat map generated from differentially expressed genes between gastrula and pluteus stages. The bar color reflects the gene expression levels. (b) Volcano plot displaying the log_2_ fold change and the −log_10_ of the *p*‐values from Kal's statistical test for the gastrula and pluteus stages. (c) Overview of log_2_ expression ratios of all transcripts differentially expressed between gastrula and pluteus stages

Quality‐filtered reads from pooled gastrulas and pluteus of *M. franciscanus* were mapped to our reference transcriptome with an ~87% mapping rate for both stages. After filtering (FDR cutoff of *p* < .05 and log_2_ fold change of ≥2), we identified 791 differentially expressed genes (DEGs; Figure 4b) from which 66.6% (519) were up‐regulated and 34.4% (272) were down‐regulated in the fully completed embryo compared to the gastrula (Table S16). Considering the number of predicted and annotated genes of *M. franciscanus* in the reference transcriptome (60,439), only ~0.5% experienced changes greater that twofold, while 95% were expressed at relatively constant levels (Figure 4b). These results may suggest that changes in a minor set of genes in the embryonic transcriptome are responsible of the morphological transition between gastrula and pluteus in *M. franciscanus*. By clustering DEGs, we obtained three main groups representing patterns of differential expression (Figure 4a,c). Some of these clusters showed similar trends (i.e., 1 and 3), but with different magnitudes, revealing increased expression in the pluteus stage in relation to the gastrula stage (Figure 4c). The most significantly up‐regulated genes were related to metallopeptidase activity (e.g., Cpa2L, Acel), metabolism (e.g., PpcL, Fpgs), calcium ion binding (e.g., Pcalr, CalmL3), adhesion (e.g., Hypp9, ThrombA1, FcolI), biomineralization (e.g., PdpiL4), germ cell nuclear factor (Gcnf1), immune system (e.g., Srcr74, Mif1), and cytoskeleton (e.g., Btub5, Fln), among others (Table S17). On the other hand, the most significantly down‐regulated genes in the pluteus stage included the sodium bicarbonate cotransporter Slc4a10, the forkhead box q2 (FoxQ2), the sulfotransferase Sult1c2, the aquaporin Aqp2, the histone ClvhH4, the t‐brain transcription factor (Tbr), the PR domain zinc finger protein 1 (Blimp1), among other genes (Table S17). Our enrichment analysis found 251 GO terms overrepresented in the 791 DEGs (Table S18), with most of them clustered in the biological process category. For gastrula, DEGs were enriched in 69 GO terms associated mainly with cell differentiation (GO:0030154) and signal transduction involved in cell cycle checkpoint (GO:0072395; Figure 5a). In the pluteus, DEGs were enriched in 182 GO terms in which positive regulation of phosphoprotein phosphatase activity (GO:0032516) was the major enriched GO term, followed by muscle contraction (GO:0006936), olfactory behavior (GO:0042048), regulation of heart rate (GO:0002027), substantia nigra development (GO:0021762), detection of calcium ion (GO:0005513), positive regulation of protein dephosphorylation (GO:0035307), and positive regulation of ryanodine‐sensitive calcium‐release channel activity (GO:0060316; Figure 5b).

**Figure 5:**
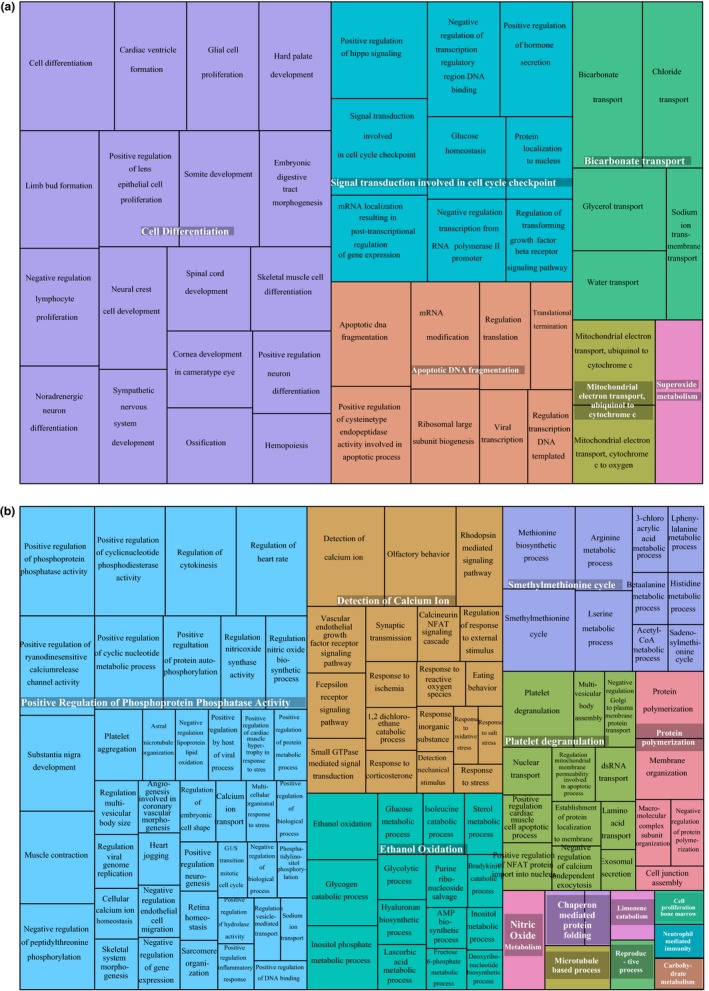
Gene Ontology treemaps for annotated differentially expressed genes. (a) Up‐regulated GO terms in (a) gastrula and (b) pluteus stages of *M. franciscanus*. When GO terms are up‐regulated in gastrula, they are down‐regulated accordingly in the pluteus

Comparisons of expression profiles in *M. franciscanus* with the well‐documented quantitative developmental transcriptome of *S. purpuratus* revealed consistent patterns among different functional classes. During the embryo‐to‐larva transition of *S. purpuratus* (30–72 hr after fertilization), more than 63% of the embryonic genes remain quiescent, while 26% reduce their levels (“transient” type) and ~11% rapidly increase their abundance (“turn‐on” type) reaching a peak during the pluteus stage (Figure 3 in Tu et al., 2014). Looking at those DEGs, we found that both species show up‐regulation of genes associated with adhesion (e.g., ThrombA1, 6Afcol, Egf/Zp, Fcol_1, Cub/Zp), cytoskeleton (e.g., Btub5, Msclact, Dynlc1‐3a, Calpn, MyX), defensome (e.g., Aldh1l1_1, Cyp2L42, Abca3a, Ugt2b11), immune system (e.g., Ubc13, lgcam, TrafB, MacpfA1, Mif4), kinase (e.g., Calm2, Camk2a_1), metabolism (e.g., Gapdh, Vha55, Lct_1, PpcL, Tkt, Tmprss5L), nervous system (e.g., AchE_7, GabrA), oogenesis (e.g., Vtgn2, YP30), among other groups (Table S19). Similarly, both species exhibit consistent profiles of down‐regulated genes for these functional classes (e.g., Sult1c_2, Hypp_2120, Vparpf_3, Fancm, MacpfB.2, Mif5, Amt1, Atp11, Aqp_2, Cycs_1, Slc4a10, Mmp14, Naalad2L, sox‐hgm, FoxQ2_1, Table S19). Despite the overall similarity between both developmental transcriptomes, several discrepancies were detected in the expression of genes from functional classes such as biomineralization (e.g., Sm30A, Msp130r6), calcium toolkit (e.g., Mlcka, Hsp701A, Pkd2), translation (e.g., VarsiB), and zing finger (e.g., Z444, Zfp509L; Table S19). These discrepancies between *M. franciscanus* and *S. purpuratus* could be explained by species‐specific differences in development (i.e., embryonic space and time), and the lower temporal resolution used our study. Nonetheless, transcriptomic comparisons during early development in other sea urchins (e.g., *Paracentrotus lividus*) have shown similar global gene expression patterns. In these organisms, complex dynamics take place during early stages before gastrulation, and major changes in the expression of gene clusters related to specialized functions (e.g., biomineralization, defensome, immunity, and nervous system) occur after gastrulation begins (Gildor et al., 2015; Tu et al., 2014).

## 4 Conclusions

Through our Illumina sequencing project, we have generated the first assembled and annotated developmental transcriptome for the giant red sea urchin *Mesocentrotus franciscanus*. This species is an important model system in fisheries research, population genetics, ecology, and climate change biology along the California Current Large Marine Ecosystem. Our early developmental transcriptome provides a detailed characterization of functional, structural, and regulatory genes involved in developmental processes, exploring their level of evolutionary conservation among echinoderms and assessing the global gene expression dynamic that underlies morphological transitions between the gastrula and the pluteus stage of *M. franciscanus*.

## Conflict of Interests

None declared.

## Data Deposition

The datasets generated and analyzed during this study are available in the National Center for Biotechnology Information (NCBI) repository into the bioproject PRJNA272924. The NCBI Sequence Read Archive codes of the raw reads are SRS823202, SRS823216, SRS823218, and SRS823221. In addition, raw reads and the assembled transcriptome have been deposited in Dryad, DOI: 10.5061/dryad.hc7v5. All other data generated or analyzed during this study are included in this published article and its supporting information files.
