# Supplementary figures and images for: Gene expression profiling during the embryo‐to‐larva transition in the giant red sea urchin Mesocentrotus franciscanus
Source: Ecol Evol. 2017 Mar 21;7(8):2798–811. doi: 10.1002/ece3.2850 (PMC5395446; doi:10.1002/ece3.2850)

Number of Blast hits

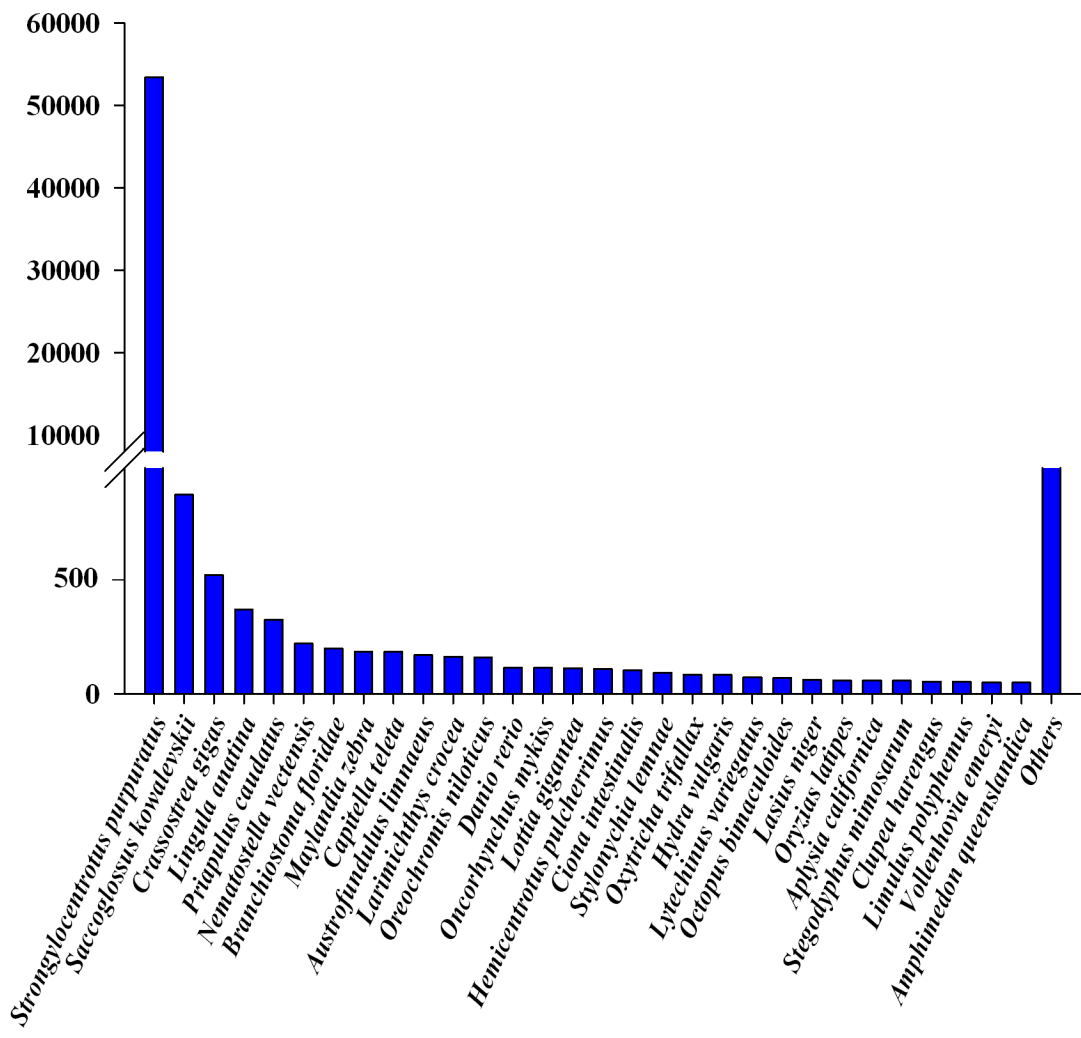

Supplement: Supplementary file 1 [file ECE3-7-2798-s001.pdf]

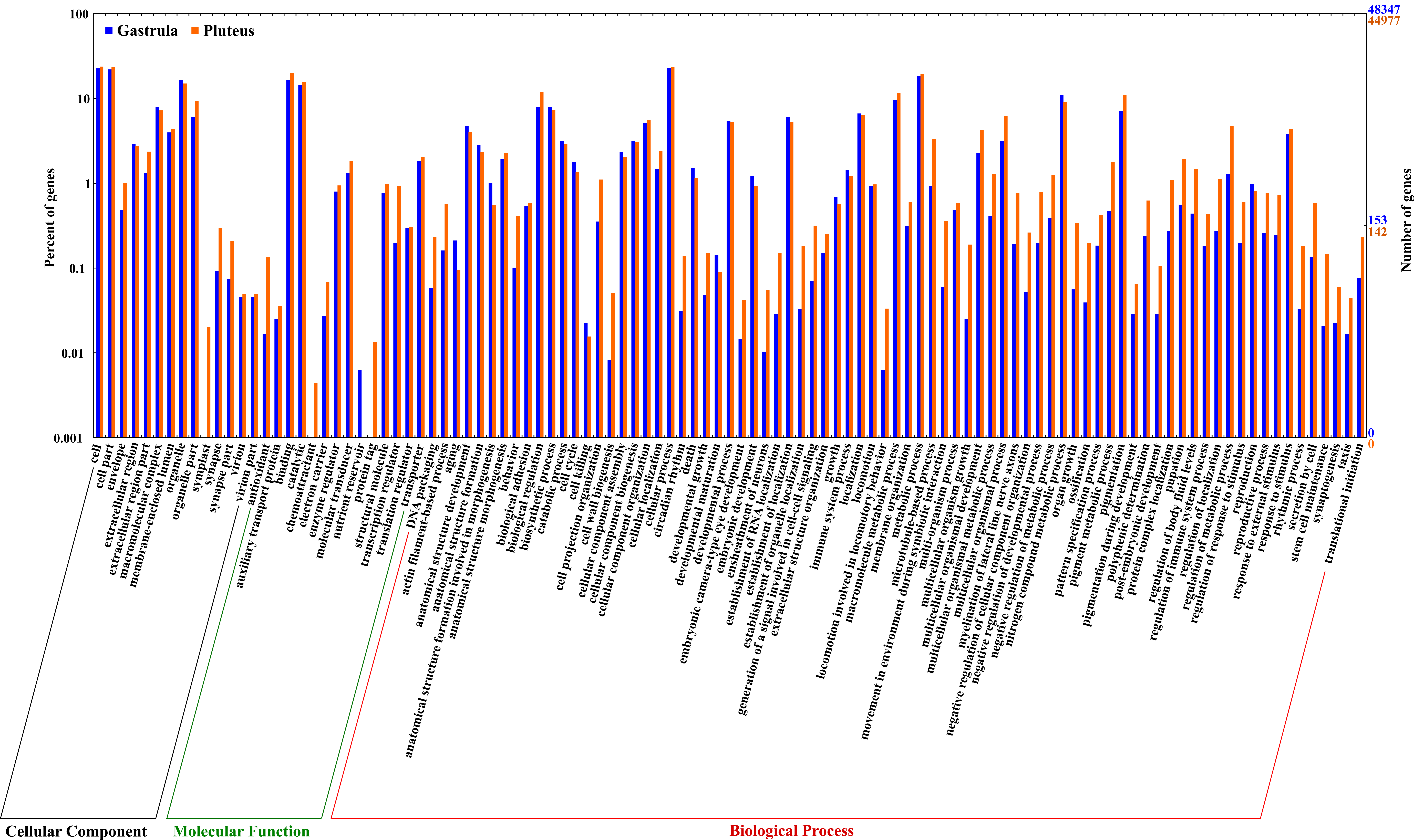

Supplement: Supplementary file 2 [file ECE3-7-2798-s002.pdf]

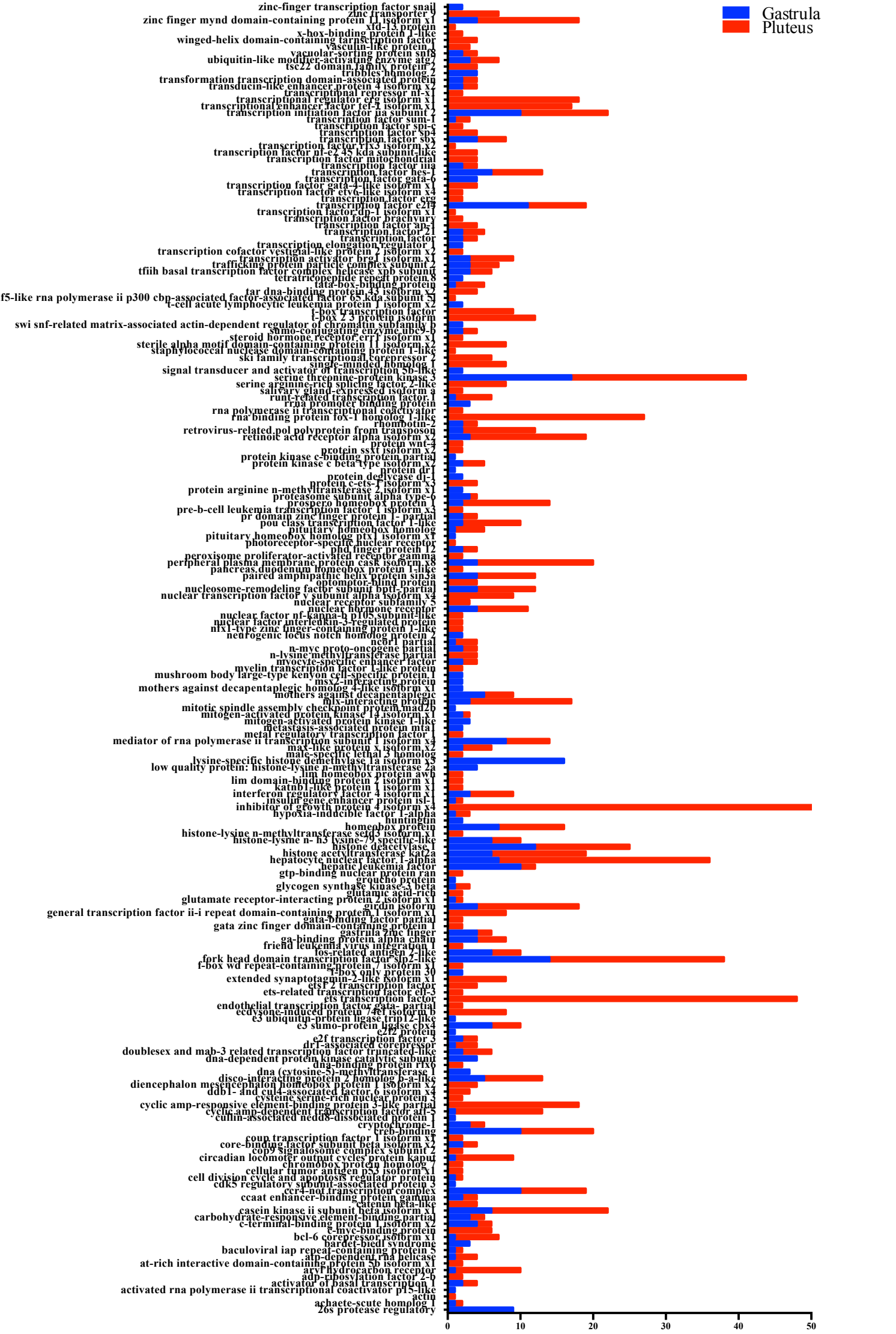

Number of genes

Supplement: Supplementary file 4 [file ECE3-7-2798-s004.pdf]
