# Supplementary material for: Gene expression profiling during the embryo‐to‐larva transition in the giant red sea urchin Mesocentrotus franciscanus
Source: Ecol Evol. 2017 Mar 21;7(8):2798–811. doi: 10.1002/ece3.2850 (PMC5395446; doi:10.1002/ece3.2850)
Supplement: Supplementary file 3 [file ECE3-7-2798-s003.pdf]

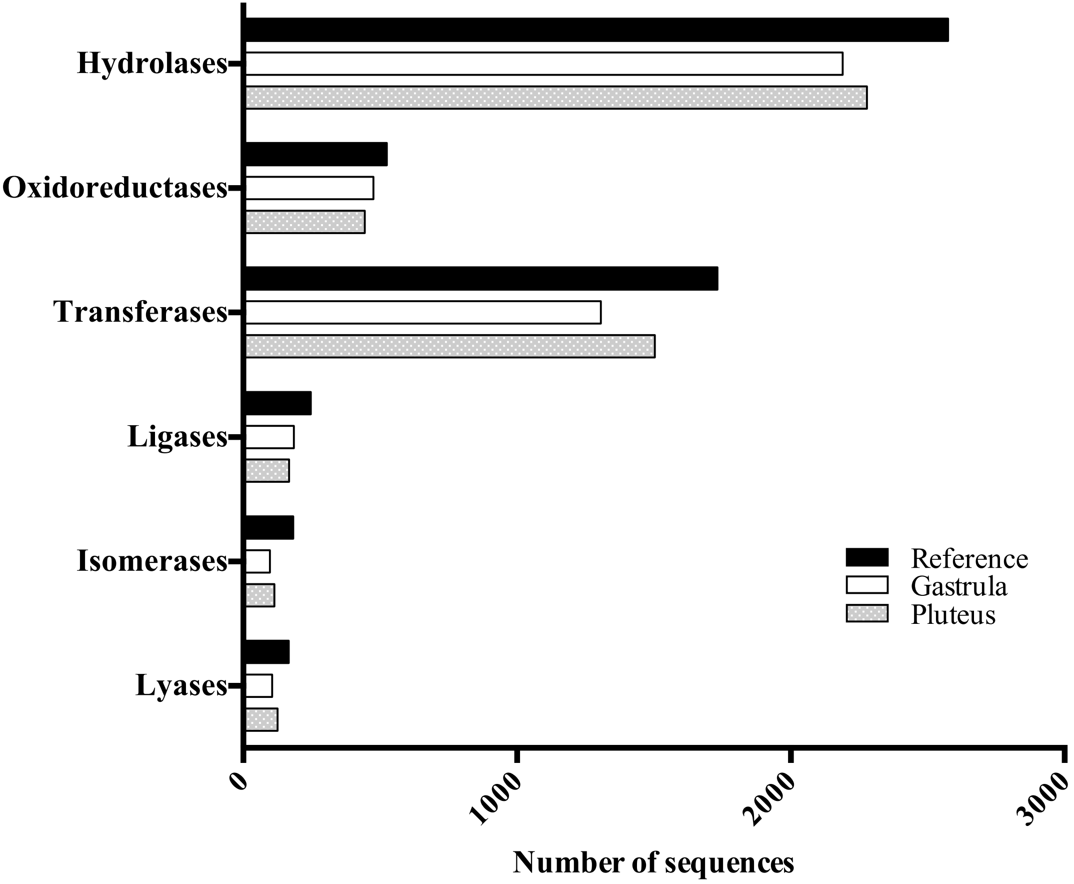

Hydrolases
Oxidoreductases
Transferases
Ligases
Isomerases
Lyases
Reference
Gastrula
Pluteus
0
1000
2000
3000
Number of sequences
